# The effects of odor and body posture on perceived duration

**DOI:** 10.3389/fnbot.2014.00006

**Published:** 2014-02-06

**Authors:** Eliane Schreuder, Marco R. Hoeksma, Monique A. M. Smeets, Gün R. Semin

**Affiliations:** ^1^Behavioural and Societal Sciences, Toegepast Natuurwetenschappelijk OnderzoekSoesterberg, Netherlands; ^2^Sensation Perception and Behaviour SSG, Unilever R&DVlaardingen, Netherlands; ^3^Department of Psychology, Faculty of Social and Behavioral Sciences, University of UtrechtUtrecht, Netherlands; ^4^Department of Psychology, Koç UniversityIstanbul, Turkey; ^5^Department of Psychology, Instituto Superior de Psicologia AplicadaLisbon, Portugal

**Keywords:** perceived duration, internal clock, arousal, affective state, body posture, odor

## Abstract

This study reports an examination of the internal clock model, according to which subjective time duration is influenced by attention and arousal state. In a time production task, we examine the hypothesis that an arousing odor and an upright body posture affect perceived duration. The experimental task was performed while participants were exposed to an odor and either sitting upright (arousing condition) or lying down in a relaxing chair (relaxing condition). They were allocated to one of three experimental odor conditions: rosemary (arousing condition), peppermint (relaxing condition), and no odor (control condition). The predicted effects of the odors were not borne out by the results. Self-reported arousal (SRA) and pleasure (PL) states were measured before, during (after each body posture condition) and postexperimentally. Heart rate (HR) and skin conductance were measured before and during the experiment. As expected, odor had an effect on perceived duration. When participants were exposed to rosemary odor, they produced significantly shorter time intervals than in the no odor condition. This effect, however, could not be explained by increased arousal. There was no effect of body posture on perceived duration, even though body posture did induce arousal. The results do not support the proposed arousal mechanism of the internal clock model.

## Introduction

Arguably, individuals can perceive the duration of a certain event differently than the actual duration of that event (Allan, 1979). A variety of interesting opportunities arise, if it were possible to influence the subjective duration of time by manipulating external factors, so that one would expand the perceived duration of enjoyment and shorten the perceived duration of unpleasant events.

Numerous studies have been conducted investigating the influence of external factors on the subjective judgment of time and different cognitive models have been developed to account for these effects^1^. Notably, external factors have been found to influence subjective time duration. How this happens appears to depend on how the timing is executed: prospectively, retrospectively or as passage of time judgments (Block and Zakay, 1997; Bailey and Areni, 2006; Wearden, 2008). In prospective time judgments, people are consciously processing time, for instance when people are told to estimate the duration of an event to come. In this situation, the internal clock model seems to apply, and subjective duration judgments are made based on an internal clock (Allan, 1979; Treisman et al., 1990; Droit-Volet and Meck, 2007). The internal clock is assumed to consist of a pacemaker that emits pulses at a given rate, the clock speed; a switch controlling gating the pulses and an accumulator, which counts the number of pulses during the event. The resulting count can be compared with a duration stored in memory and a duration judgment can be made. Thus, the more pulses gated to the accumulator the longer the perceived duration. Two mechanisms are proposed that could increase (or decrease) the number of pulses accumulated and lead to an increase (or decrease) of duration judgments (see Wittmann and Paulus, 2008 for a graphical representation of the internal clock model).

First, increased attention to the processing of time leads to an accumulation of more pulses; it closes the switch leading the accumulator to start counting pulses. When attention is driven away from time processing (for instance by external factors like music), the switch is opened and fewer pulses are accumulated. As a result the duration is judged shorter. This is the attention mechanism of the internal clock model. Second, arousal (i.e., readiness for action) is assumed to increase the rate at which the pacemaker generates pulses leading to a faster accumulation of pulses over time. Thus, a time interval will be perceived as longer when a person is aroused, because more pulses are generated and accumulated compared to a non-aroused person (Wittmann and Paulus, 2008).

In retrospective timing judgments, however, when people are not consciously processing time, due to a preoccupation with non-temporal tasks, or because they were not told to time the duration, the effects of external factors on perceived duration are better described by discrete events models (Ornstein, 1969; Block, 1985; Kellaris and Kent, 1992; Ivry and Spencer, 2004; Bailey and Areni, 2006; Khoshnoodi et al., 2008). These models posit that the duration of a target period is reconstructed from non-temporal information (Bailey and Areni, 2006; Khoshnoodi et al., 2008), stored memories (Ornstein, 1969) or number of working memory updates (Ivry and Spencer, 2004) associated with the interval in memory. More discrete events during the target interval result in a longer estimate of that interval. For instance, Block and Reed (1978) required subjects to encode word lists at different levels of processing. One group judged the typing style of words (a *shallow* task), another group categorized words into semantic categories (a *deep* task), and a final group alternated between both tasks. Afterward, all of the subjects were unexpectedly asked to judge which activity seemed longer. The attention mechanism of the internal clock model would predict that the shallow task would have been perceived longest, because the least attention is distracted from time processing. However, it was found that participants perceived the alternated shallow-deep strategies to last longest (i.e., most discrete events). Bailey and Areni (2006) also attributed the fact that retrospective duration judgments of familiar music compared to unfamiliar music is longer to the discrete events model. They argued that more elements are remembered when music is familiar. At last, Wearden (2008) distinguishes a third timing type, passage of time judgments, where people are not asked how long an event lasted, but how quickly time seemed to pass during it (as normal, more quickly or more slowly, for instance). Wearden (2008) argues that passage of time judgments are systematically effected by hedonic impressions of the event, whereas retrospective judgments seem unsystematically related. However, no explaining mechanisms are proposed.

In the current study, we focus on prospective timing and the internal clock model. Numerous empirical studies support the internal clock model and the attention mechanism (Hornik, 1984; Tremblay and Fortin, 2003; Droit-Volet et al., 2010), however empirical evidence supporting the speeding clock effect induced by arousal is mixed. The research supporting this model stems from studies that administered drugs to increase arousal or that provided repetitive series of auditory clicks or visual flickers before an event that had to be timed. These studies found longer duration judgments when arousal level was increased (Meck, 1983; Wearden and Penton-Voak, 1995; Droit-Volet and Wearden, 2002). More complex results stem from Angrilli et al. (1997), who did not find a main effect of arousal on perceived duration. However, they found an interaction between valence and arousal. Participants underestimated low arousal negative images and high arousal positive images, and overestimated low arousal positive images and high arousal negative images. They argued that in the low arousal condition the negative images distracted attention away from time processing and as a result duration was perceived as being shorter, while in the high arousal negative condition people activate their defensive system and prepare to fight or flight. The latter is associated with an increase in internal clock speed, which increases the duration judgment. However, there are also conflicting results. Vroon and van Boxtel (1972) found that duration estimation curves did not differ before and after administration of drugs stimulating arousal. Noulhiane et al. (2007) found an opposite effect of arousal. They reported that people underestimated the duration of high arousal sounds more consistently compared to low arousal sounds. As an explanation Droit-Volet and Meck (2007) argued that in the high arousal condition, attention was distracted from the processing of time that in turn resulted in an underestimation of duration. Tipples (2010) proposed that the effect of arousal on perceived duration depends on the sensory modality that induced the arousal (ears vs. eyes). Thus, the influence of arousal on perceived duration is not as straightforward as the model proposes. In sum, whether arousal is able to influence time processing seems to depend on the valence of the arousal, the ability to distract attention from time processing or perhaps even on the sensory modality that induced the arousal. Therefore, in the present study we aim to extend the empirical evidence on the arousal mechanism proposed by the internal clock model.

We investigated the effect of odor and body posture on perceived duration. Odor and body posture were selected because of their arousal inducing qualities. According to Bensafi et al. (2002) and Seubert et al. (2009) odors can provoke explicit changes in level of arousal. Furthermore, Dalton et al. (2008) have developed categorizations of odors based on the common responses they elicit in terms of activation, potency, and pleasantness. Theories of embodied cognition suggest that manipulations of body posture affect the way emotional information is processed (Niedenthal, 2007). The hypothesis that body posture affects arousal level is supported by the preliminary results of Elliott et al. (2005) who suggested that changes in position may have a significant impact on behavior. They observed significantly more behavior in the standing position as opposed to a supine position, which indicates a higher level of arousal in the standing position.

To our knowledge no previous study has been conducted on the effect of odor and body posture on perceived duration. According to the arousal mechanism of the internal clock model, if arousal induced by odor and body posture increases the internal clock speed, then this will result in a overestimation of the duration. This hypothesis was tested in a time production and a password memory task. Note that in time production tasks, participants have to indicate when they think a certain time span has elapsed, for example by pressing a key. Overestimation of duration leads to shorter produced intervals: when an on-going duration is subjectively judged to be longer, the end of the interval production is produced earlier to match the duration to be produced. Time productions are therefore shorter when duration is overestimated (Tremblay and Fortin, 2003). Thus, we expected time productions to be shorter in the high arousing compared to the low arousing conditions of our experiment. Previous research (e.g., Penton-Voak et al., 1996) also mention a slope effect when the internal clock speed is sped up, meaning that the effect of the manipulation must be larger at longer durations. The aim of our study was not to prove this acceleration of the clock itself but rather whether we could find the symptoms of an accelerated clock first. Therefore the experiment was not specifically designed to be able to find a slope effect and as a result we had no expectations to find a slope effect.

## Materials and methods

### Participants

Sixty students (45 females, 15 males) from the University of Utrecht participated in this study. They were between 18 and 35 years (Mean age 22.2 ± 3.4 years).

### Experimental design

Participants conducted a time production task while sitting upright (arousing condition) or lying down in a relaxing chair (relaxing condition), while being exposed to an odor. The independent categorical variables were: Odor, Body posture, and Interval. The dependent variables were Time production and Arousal.

#### Independent variables

Odor was a between-subject factor with three levels, namely Relaxing, Arousing, and Control (no odor). In three pilot studies preceding the experiment we tested the arousal properties of a number of odors, namely rosemary, citrus, peppermint, and the potential relaxing properties of vanilla and lilial. Perceived pleasantness of the odors was also tested. We used a between-subject design (in line with the set-up of the main experiment). Average arousal scores (scale 1–9) were highest for the rosemary odor [Mean = 5.8 (*N* = 16)] and lowest for the peppermint odor [Mean = 4.7 (*N* = 11)]. This was an unexpected finding as peppermint is generally considered to be alerting (Warm et al., 1991). However, results reported by Dalton et al. (2008) also showed that rosemary was perceived as arousing and that peppermint scored low on activation. Activation is related to arousal level in our study and appears to correspond to the results of our pilot study. The pilot also revealed that both rosemary and peppermint were perceived as pleasant, which was another criterion as odor valance could impact perceived duration differently. Thus, based on these results, we decided to use a rosemary odor (50% v/v in Iso-propyl-myristate (IFF, International Flavors and Fragrances inc., Hilversum, NL) to induce arousal and a peppermint odor (50% v/v in Isopropyl-myristate: IFF) to induce relaxation (i.e., less arousal).

Body posture was a within-subject factor with the levels: Arousing chair and Relaxing chair. We used a normal office chair in upright position for the arousing condition. A relaxing, reclining chair with an almost horizontal position was used in the relaxing condition.

Interval was a within-subject factor with the levels: 1.33, 1.58, and 2.17 min. These three time intervals needed to be produced in each time production task. The main selection criterion was that we wanted to use intervals that exceeded one minute, as these seem harder to produce because participants need to concentrate for a longer period of time. To motivate the participants to be as accurate as possible, we did not use round numbers. To avoid order effects, the time production intervals were randomly presented to the participants. In each time production a different password needed to be remembered. We used the following passwords in the arousing chair condition: Qp5y3Djm, Z2Hx89bS, TFr7L4Vg. In the relaxing chair condition the following passwords were used: MbUE4ZkC, T8FxJ2gL, W13sA1nZ. Passwords were presented randomly. A pilot test revealed that the used passwords were rated equally difficult. In this sense perceived difficulty of the memory task was equal for all conditions and did not influence the time production tasks differently.

#### Dependent variables

The dependent variable was time production measured in seconds of over- or underproduction (produced time – indicated time). Thus, negative values indicate underproduction and positive values indicate overproduction. Participants executed a duration production task in which they had to estimate a given time interval by pressing a mouse button when they started the time production and by clicking the mouse again when they thought the given time had elapsed. To avoid counting, an 8-digit password needed to be remembered simultaneously. This method was chosen because a duration production task could be used to explain individual differences in terms of attention mechanism or the clock speed. This is not the case in duration reproduction (a standard interval with standard duration is presented. Subsequently, participants have to reproduce the length of this interval by indicating when they believe that the duration is now identical to the standard interval) or duration discrimination tasks (two intervals are presented and participants have to decide which one is longer). Any internal influence by arousal or attention would affect both intervals (standard and comparison) and thus would not reveal differences in outward performance.

In order to check whether odor and body posture influenced arousal level, average heart rate (HR) and skin conductance response (SCR) as well as self-reported level of arousal (SRA) and pleasure (PL) were recorded. An affect grid (Russell et al., 1989) was used to measure self-reported level of arousal and PL. The grid consists of two 9-point scales ranging from feeling aroused to sleepy and from feeling pleasant to unpleasant. A score of 5 indicates a neutral score. HR and SCR were recorded with Biopac amplifiers (Biopac Systems Inc., USA). A photoplethysmographic ear-clip attached to the earlobe was used to assess HR. SCR was measured by electrodes that fitted around the tip of the middle finger and ring finger and that were attached via Velcro straps. Sampling rate was 1000 Hz for both measures. SCR amplitude tends to increase with increasing levels of arousal, be it positive or negative (Bensafi et al., 2002). HR also increases with increasing arousal levels (Fowles, 1980). To record baseline values, we measured SRA, PL, HR and SCR before the experiment. Subsequently, we measured SRA and PL after and HR and SCR during the experimental task.

### Procedure

The experiment was conducted at the University of Utrecht and was approved by the Ethics Committee of Faculty of Social and Behavioural Sciences of the University. Before the experiment started, each participant received an information letter with the instructions for the experiment. In case of queries further instructions were given orally. Watches and telephones were handed in, so these could not be used during the experiment. After the instructions, the participant had to fill out and sign the informed consent form. This form also indicated the participant's number and whether the participant started with part 1 (time production task in the arousing chair) or part 2 (time production task in the relaxing chair).

After signing, a resting period of 10 min was included, in which a demo of the experimental task was shown. The participant also had to fill out the affect grid. After the resting period, baseline measures of HR and SCR in rest were recorded for three minutes in the control (no odor) room.

Subsequently, each participant was pseudo randomly (as groups were matched) assigned to a specific room. Three rooms were used: one room with a rosemary odor, one room with a peppermint odor and a control room with no odor. Special pellets were used to control the odor intensity in the experimental rooms. These pellets absorb the diluted odor oil and release the odor with a fixed intensity during the course of the day. We used five pellets per day for rosemary as well as peppermint, with an average evaporation rate of about 0.01 gram of odorant dilution per 15 min. Twenty participants were assigned to each room, resulting in 20 participants in each experimental odor group. The groups were matched for age and sex (mean age control odor: 21.8 ± 2.3; peppermint 22.1 ± 4.2; rosemary 23.1 ± 2.5). This was done because age and sex might influence perceived duration (Droit-Volet et al., 2007; Grassi, 2010; Hancock and Rausch, 2010).

Once seated in a room (either in the relaxing or arousing chair, depending on the part the participant started with), the participant was connected to the Biopac bio-amplifiers to measure HR and SCR during the experimental task. After this, the time production task was started on a computer (three computers were used, one in each room). The participant had to enter the participant number and whether he or she started with part 1 or part 2 (as indicated on the informed consent form). A welcome screen was presented, and once the participant pressed a mouse button the memory task started.

A password that was to be remembered was displayed for 15 seconds. Fifteen seconds was chosen because that was long enough to really remember the relatively difficult password. After these 15 seconds the time production task started with a screen on which the time interval to be estimated appeared. The participant's task was to accurately estimate the interval by clicking the mouse twice, once for onset and once for offset. The screen indicating the to-be-produced interval was shown infinitely, until the participant pressed a mouse button. This was done to make sure that the participant made a conscious time production as he or she exactly knew when the timer started. After the button was pressed, the screen turned white. When the participant believed the required time interval had expired, he or she clicked the mouse again. An input field appeared where the participant could enter the remembered password. After the password was submitted and the participant pressed ENTER (indicated on the screen), a new password appeared and the next trial with a new time interval started. E-prime (Psychology Software Tools, Sharpsburg, USA) was used to present and record the time production and password memory task. After the third trial the participant was requested (via the computer screen) to fill out the affect grid and, subsequently, to leave the room. During this period the experimenter changed the chair and the corresponding time production task was again started on the computer, and the procedure was repeated. When the last password was submitted a goodbye screen appeared. The screen requested the participant to fill out the arousal grid again, and to leave the room afterwards. Thus, the time production task, consisting of three time intervals, was conducted twice; once in the arousing chair (part 1) and once in the relaxing chair (part 2). Body posture was counterbalanced to avoid order effects. Therefore, half of each experimental group started the experiment in part 1; the other half started in part 2.

Outside the experimental room the participant received a debriefing. In the debriefing we asked questions whether the participant understood the aim of the experiment, and if and how the odor was perceived, so as to be able to understand possible (trigeminal) effects of the odors, and to be able to investigate how the different odors were perceived. Furthermore, participants in an odor group who did not perceive an odor were tested whether they were able to smell. Participants received 1 course credit for participating in the experiment or a remuneration of 5 euro.

### Statistical design

Main and interaction effects of Odor, Body posture and Interval on time production were analyzed with a 3 × 3 × 2 repeated measures mixed design ANOVA (general linear model). Alpha was set at 5%. To investigate significant differences in physiological and SRA measures during baseline, the relaxing and arousing conditions, repeated measures ANOVAs (mixed design) were carried out. Pearson correlation tests were carried out to investigate correlations between the arousal measures and time production scores. Additionally, we investigated whether Odor and Body posture had a direct effect on time production, an indirect effect via arousal, or have no effect at all using linear regression.

## Results

### Sample data

Six time productions were made by every participant. It appeared that some participants did not seriously produce the requested time intervals. We decided to exclude the cases that showed extreme time productions for four or more time intervals, which was interpreted as meaning that the extreme score was not an incident. Extreme scores were defined as exceeding the mean by two standard deviations (e.g., a participant reported 10 s for a 1.33 min interval). We decided to leave the extreme score in the dataset if a participant showed an extreme time production only once or twice. Four cases were excluded using these criteria. One additional case was excluded due to missing data. Table 1 shows the resulting sample sizes of the experimental groups.

**Table 1:** **Sample size experimental groups**.

**Odor group**	**N**
Control	17
Peppermint	19
Rosemary	19

The main and 2nd order interaction effects of odor, body posture, and interval on time production were analyzed and are reported in de following sections.

### The effect of odor, body posture, and interval on time production

A repeated measure ANOVA with a mixed design was carried out to analyse the effect of Odor, Body posture, and Interval on time production. We expected a main effect of Odor and Body posture. We expected no effect of Interval or second order interaction effects. Mauchly's test indicated that the assumption of sphericity was met. Tables 2, 3, 4 show the estimated mean over- and underproduction scores for the Odor groups, Body postures, and Intervals respectively.

**Table 2:** **Estimated mean over- and underproduction scores in seconds**.

**Odor group**	**Mean**	**Std. error**
Control	9.835	7.043
Peppermint	−6.490	6.662
Rosemary	−18.011	6.662

**Table 3:** **Estimated mean over- and underproduction scores in seconds**.

**Body posture**	**Mean**	**Std. error**
Arousing chair	−5.613	4.427
Relaxing chair	−4.165	4.261

**Table 4:** **Estimated mean over- and underproduction scores in seconds**.

**Interval**	**Mean**	**Std. error**
1.33	−0.300	3.585
1.58	−8.330	4.451
2.17	−6.037	5.350

As expected, we found a main effect for Odor on time production *F*_(2, 52)_ = 4.144, *p* = 0.021.

Table 2 shows that the rosemary group had the highest underproduction scores. The peppermint group also under-produced time, but not as much as the rosemary group. The control group (no odor) tended to over-produce time. Bonferroni pairwise comparisons were conducted. One-tailed tests show that the participants in the rosemary group produced shorter time intervals than participants in the control group (*MD* = −27.846, *p* = 0.009). There was no significant difference between the rosemary and the peppermint group or between the control and the peppermint group.

**Figure 1:**
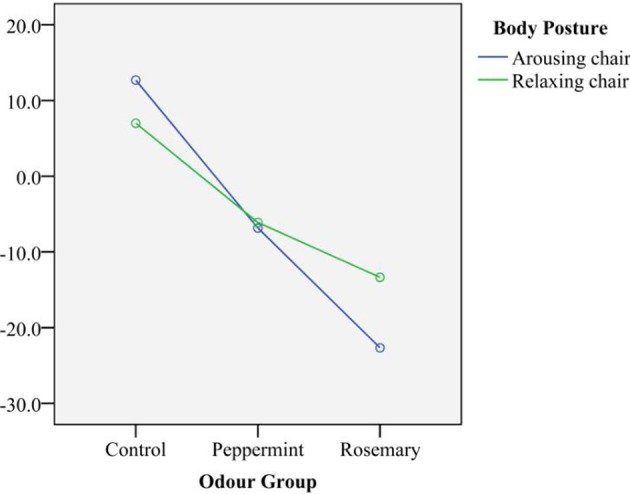
**Over-underproductions in seconds per odor group and body posture**.

Against our expectation, we found no main effect of Body posture on time production. As expected, there was no main effect of Interval and we found no second order interaction effects between Odor, Body posture, and Interval on time production. Figure 1 depicts the Odor and Body posture effect on time production.

**Figure 2:**
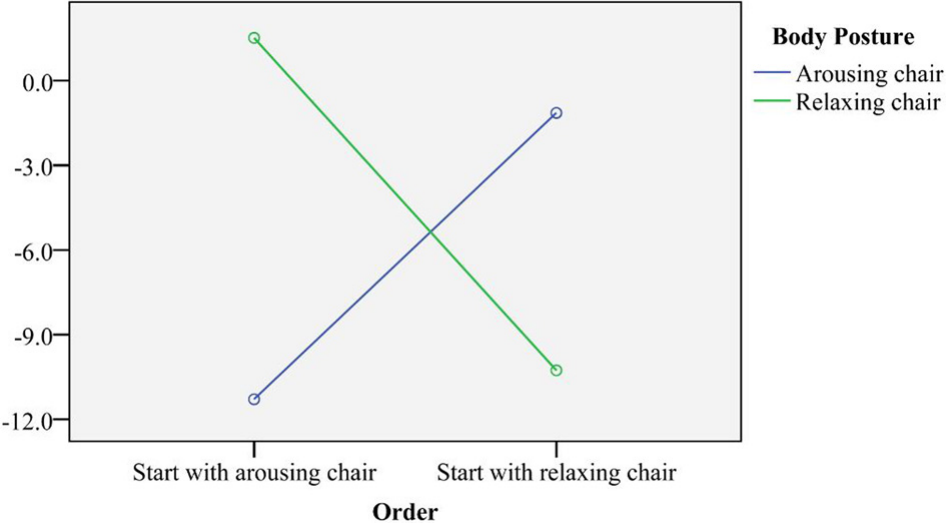
**Interaction effect between body posture and order on time production in seconds.** Note that the data point arousing chair and start with relaxing chair means that the group did the experiment in the arousing chair after they did the experiment in the relaxing chair (as they started with the relaxing chair). The data point arousing chair and start with arousing chair indicates that the group started the experiment in the arousing chair and, subsequently, they did the experiment in the relaxing chair (data point: relaxing chair, start with arousing chair).

As a next step, we included Order in the model. This was done to investigate if it mattered whether participants started the experiment in the arousing or the relaxing chair. We expected no main effect of Order on time production nor second order interaction effects with Odor or Body posture. A repeated measures ANOVA showed no main effect of Order or an interaction effect between Order and Odor on time production. Interestingly, an interaction between Body posture and Order on time production was found *F*_(1,49)_ = 9.613, *p* = 0.003 (see Figure 2).

The interaction effect shows that participants tended to under-produce time in the chair where they started the experiment by on average 10.8 s. When participants did the experiment again in the other chair, they tended to increase time productions and became more accurate (on average 0.2 s underproduction), regardless of the body position in that chair, therefore a learning effect appears to have taken place.

#### The effect of arousal

The first question we asked was: “did Odor and Body posture affect arousal?” Subsequently, we controlled the effect of Odor and Body posture on time production for arousal and investigated the effect of arousal on time production.

#### Arousal manipulation

***Self-reported arousal (SRA) and pleasure (PL)***. A repeated measures ANOVA was carried out to test whether Odor and Body posture induced SRA and PL as indicated on the affect grid. Mauchly's test indicated that the assumption of sphericity was met. We found a main effect of Body posture on SRA *F*_(2, 114)_ = 12.042, *p* < 0.001 and PL *F*_(2, 114)_ = 4.261, *p* = 0.016. Bonferroni pairwise comparisons showed that doing the time production task in the arousing chair (Mean = 5.45) was reported as more arousing than in a relaxing chair (Mean = 4.42; *MD* = 1.033, *p* < 0.001, one-tailed test) as expected. The relaxing chair was perceived as more pleasant (Mean = 6.32) than the arousing chair (Mean = 5.73; *MD* = 0.583, *p* = 0.010). Baseline SRA scores in the arousing chair (Mean = 4.98) were higher compared to scores in the relaxing chair, but lower than those in the arousing chair during the experiment. These differences were not significant, but a trend was identified (*MD* = 0.567, *p* = 0.062; *MD* = −0.467, *p* = 0.061 respectively). There were no main effects of Odor on SRA or PL, nor did we find interactions between Odor and Body posture on SRA or PL.

***Skin conductance response (SCR)***. The effects of Odor and Body posture on SCR (measured by average SCR amplitude in microsiemens) were tested with a repeated measures ANOVA. Due to technical problems, nine cases in the rosemary group and four cases in the control group were excluded. Mauchly's test indicated that the assumption of sphericity had been violated [χ^2^_(2)_ = 9.778, *p* = 0.008], therefore degrees of freedom were corrected using Huynh-Feldt estimates of sphericity (ε = 0.899). We found a main effect of Body posture on SCR *F*_(1.80, 79.10)_ = 25.546, *p* < 0.001. There was no effect of Odor or an interaction effect between Odor and Body posture on SCR. Bonferroni pairwise comparisons showed that SCR in the arousing chair condition (Mean = 6.79) was significantly higher than in the relaxing chair condition (Mean = 6.37; *MD* =.414, *p* = 0.036, one-tailed test), as expected. Furthermore, during the baseline (Mean = 5.22) SCR scores were lower than during the time production tasks in any position (*MD* = −1.562, *p* <.001; *MD* = −1.148, *p* < 0.001; respectively). Apparently, SCR was more sensitive to whether or not a task was executed than to body posture. However, SCR scores did indicate that arousal was indeed induced by manipulating body posture during the experiment. Odor did not influence SCR significantly.

***Heart rate (HR)***. A repeated measures ANOVA was carried out to test whether Odor and Body posture induced arousal as measured by average HR in beats per minute. No cases were excluded in this analysis. Mauchly's test indicated that the assumption of sphericity had been violated [χ^2^_(2)_ = 22,562, *p* < 0.001], therefore degrees of freedom were corrected using Huynh-Feldt estimates of sphericity (ε = 0.794). We found a main effect of Body posture on HR *F*_(1.59, 90.50)_ = 4, 146, *p* = 0.027 but no effect of Odor or interaction effect between Odor and Body posture on HR. Bonferroni pairwise comparisons showed that, as expected, HR was significantly higher when sitting in an arousing chair during the experiment (Mean = 89.99) than when sitting in a relaxing chair (Mean = 84.21; *MD* = 5.780, *p* = 0.016, one-tailed), indicating that the former induced higher arousal levels. Furthermore, the results showed that HR was higher during baseline (Mean = 93.77) than in the relaxing chair (*MD* = 9.559, *p* = 0.048, two-tailed), indicating that the relaxing chair induced relaxation. Odor did not influence HR significantly.

To summarize, we found that body posture induced arousal as expected, measured by the objective (HR, SCR) as well as the subjective arousal (SRA) measures. Therefore we concluded that all measures indeed measured arousal. No effects of odor were found on any measure of arousal.

#### The effect of arousal on time production

The previous sections showed that body posture induced arousal, although it did not influence time productions. Furthermore, we found an effect of odor on time production but no significant effect of odor on arousal. These results imply that the odor effect on time production cannot be explained by arousal and that arousal did not affect time production. In order to explore this idea we generated a Pearson's correlation matrix investigating possible correlations between all arousal measures, PL scores, and time production. To conduct these tests, we calculated the average increase or decrease in arousal and PL by subtracting the baseline of each experimental score. This resulted in an arousal/PL score per measure (SRA, SCR, HR, PL) per experimental part. Thus, SRA1 means the increase/decrease in SRA in the arousing chair (part 1), SRA2 means the increase/decrease in SRA in the relaxing chair (part 2) etc. Moreover, we averaged the over- and underproduction scores for each part, resulting in a single average time production (ATP) score per part. Between these arousal and PL measures, we found significant correlations between HR1 and SCR2 (*r* = 0.285, *p* = 0.047), PL2 and SCR1 (*r* = 0.318, *p* = 0.029), PL2 and SCR2 (*r* = 0.333, *p* = 0.020) and between PL1 and SCR1 (*r* = 0.294, *p* = 0.045). There were no significant correlations between objective and SRA measures. The latter indicates that a physiologically aroused person might not perceive arousal and vice versa.

We found no significant correlations between arousal scores and time productions. In order to examine whether the odor effect could be explained by arousal level, we decided to control the effect of odor and body posture on time production for arousal by including arousal indicators (HR1,2 and SRA1,2) as covariates. HR was chosen as objective arousal measure because this measure was not confounded with PL, like SCR. We found no significant effects of the arousal indicators on time production. Furthermore, the arousal indicators did not affect the odor effect; the effect became even more significant *F*_(2, 48)_ = 5.209, *p* = 0.009). Thus, the odor effect on time production was not driven by arousal.

Interestingly, we found a significant correlation between PL scores in the relaxing chair and average time productions (*r* = −0.307, *p* = 0.023); shorter time productions in the relaxing chair were associated with more PL. Linear regression showed that 9% of the variability in time production scores in the relaxing chair was explained by the variability of PL scores (*R*^2^ = 0.094, *F* = 5.515, *p* = 0.023). Additionally, we tested whether the odor effect could be explained by different PL scores. The results of the ANOVA showed that PL in the relaxing chair had a significant effect on time production *F*_(1, 51)_ = 4.381, *p* = 0.041. The odor effect remained significant too *F*_(2, 51)_ = 3.872, *p* =.027. This means that the odor effect could not be explained by PL. Thus, time production is influenced by odor as well as PL but it is not affected by arousal.

**Figure 3:**
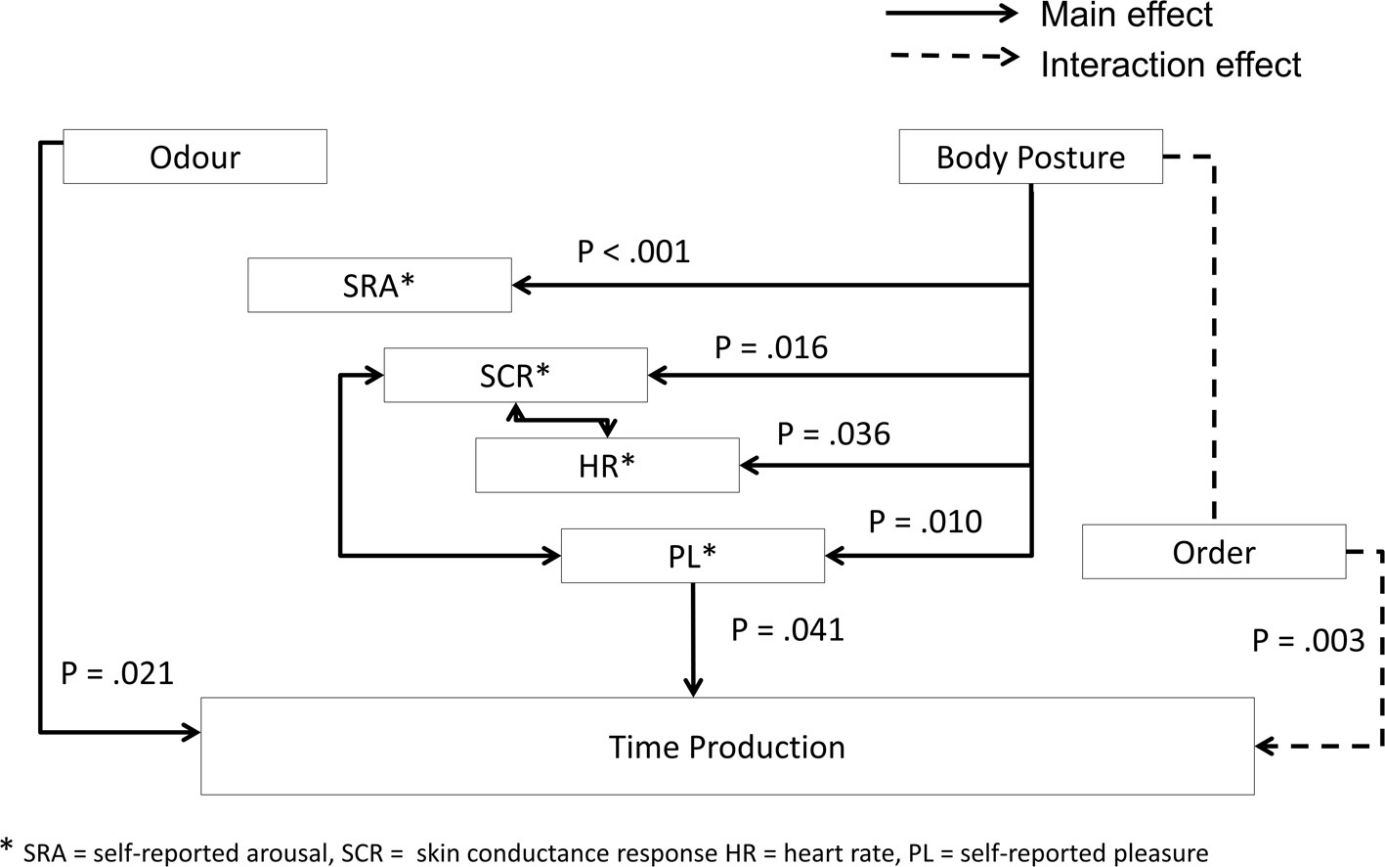
**Overview of the results**.

Figure 3 provides an overview of all results, *p*-values of effects (*p* < 0.05) are indicated.

## Discussion

The aim of this study was, firstly, to generate additional insights into how external factors can influence subjective time duration and secondly, to find further empirical evidence for the arousal mechanism of the internal clock model. We investigated the effect of odor and body posture on time production and expected that both factors would influence perceived duration via the arousal mechanism as proposed by Treisman et al. (1990). According to the arousal mechanism, increased arousal speeds up the internal clock and as a result duration is overestimated and time productions are shortened.

We found that odor influenced perceived duration. Participants who were exposed to a rosemary odor produced significantly shorter time intervals than participants who were exposed to no odor. No effects of body posture or interactions between body posture and odor on perceived duration were found. Although we found the expected odor effect on perceived duration, we found no support that the effect was caused by increased level of arousal. Firstly, the physiological arousal measures as well as the self-reports did not differ between the control and odor groups. This indicates that odor was not able to induce arousal during the experiment. A correlation matrix showed that arousal levels did not correlate with time productions. Secondly, when controlling for arousal, the odor effect on perceived duration remained significant, indicating that the odor effect is not explained by arousal.

It could be argued that arousal measures were invalid, and therefore no effect was found. This claim can be refuted as we found that body posture induced arousal but had no effect on perceived duration. Both the physiological arousal measures and the SRA measure showed that participants felt more aroused in the arousing chair compared to the relaxing chair. Therefore, we assume that the arousal measures were valid. Based on these findings we conclude that the odor effect on perceived duration was not caused by increased arousal and that arousal induced by body posture has no effect on perceived duration.

Thus, in this study we found no support for the arousal mechanism of the internal clock model. An explanation could be that the produced time intervals were too long to be affected by arousal. Other research assessing the effects of arousal on time perception usually investigate intervals up to very few seconds (Droit-Volet and Meck, 2007; Gil and Droit-Volet, 2009), which makes them hard to compare with this study. Angrilli et al. (1997) found a temporal overestimation of high-arousal negative pictures when these were shown for two seconds, but not for longer durations. This suggests that the arousal effect on perceived duration is rather short and autonomous. Meissner and Wittmann (2011) found a relation between HR and time reproductions up to 20 s. As our time production intervals all exceeded one and a half minute and are beyond the reported time intervals affected by arousal, other mechanisms might have been in place. Angrilli et al. (1997) suggested that attention related mechanisms presumably prevail at longer durations. Following the attention mechanism, our results can be seen to suggest that people in the rosemary condition were the least distracted and focused more on the time processing task (more time accumulated, which leads to shorter productions as time is overestimated).

To tentatively test this theory, we reasoned that if the memory task distracted participants from time processing, password recall accuracy could be expected to be a mediating factor in the odor effect. We expected that the higher the password accuracy, the more attention was paid to the memory tasks, the more distracted the participant was and the longer the time productions would be. Table 5 reports the average password accuracy scores per Odor group and for every Part. Twenty-four points could be earned by a correct recollection of the password. Errors resulted in a reduction of points. Accuracy was calculated as the percentage of digits correctly recollected taking also position and capitals into account. Note that a participant made six recollections (three per part).

**Table 5:** **Mean password accuracy**.

**Odor group**	**Part**	**Mean (%)**	**Std. deviation**	***N***
Rosemary	1 (arousing)	82	0.245	60
	2 (relaxing)	82	0.209	60
	Total	82	0.227	120
Peppermint	1 (arousing)	88	0.152	60
	2 (relaxing)	86	0.212	60
	Total	87	0.184	120
Control	1 (arousing)	89	0.126	60
	2 (relaxing)	85	0.167	60
	Total	87	0.148	120
Total	1 (arousing)	86	0.183	180
	2 (relaxing)	85	0.197	180
	Total	85	0.190	360

According to Table 5 the rosemary group performed worse than the other odor groups, which could indicate that they put less effort in the memory task leading to longer time estimations (more focused on time processing) and shorter productions. We ran a longitudinal multilevel analysis [for more detail on the analysis see Hox (2010), chapter 5] to test this hypotheses. Occasion (chronological trials), Odor, Body posture, and Password accuracy were the independent variables included in the model. Time production (represented in over- and underproductions) was the dependent variable. We found an effect of Occasion (*p* < 0.001) on Time production, which is comparable to the interaction effect found between Order and Body posture (see next paragraph), and an effect of Odor (*p* = 0.26). We found no effect of Body posture or Password accuracy. Thus password accuracy was not a mediating factor. As we found no interference of password accuracy and we might have used too long time intervals to be applicable to the internal clock model, discrete event models might better explain the effects reported here.

Since password accuracy was quite high (85% correct on average), it could be argued that the discrete events model applied, because the memory task occupied the participants too much. Rattat and Droit-Volet (2011) showed that an interference task distorted time perception more strongly than other “no counting” strategies, and as we used an interference task perhaps more retrospective timing strategies were applied. Additional analyses were conducted to test this assumption. Based on the debriefing we found that 80% of the rosemary group noticed, but not recognized the odor, and that only 45% of the peppermint group noticed the odor but all recognized it. This could explain why peppermint had no significant effect on perceived duration, as the peppermint odor was hardly noticed. Moreover, it appeared that none of the participants who had noticed the rosemary odor could identify the odor. Although in a different setting, Degel and Köster (1999) investigated the effect of odor identification on performance and implicit memory. They found that people who could not identify the odor built up new episodic memories for that odor, however those who did identify the odor did not store new episodic odor experiences. Similarly, the fact that the participants in our study could not identify the rosemary odor might have had an effect on the time production task. Due to the fact that rosemary could not be identified, new memory updates were made and as a result duration was overestimated and time productions shortened. Further research in a controlled setting is necessary to confirm this finding.

As mentioned above, we found an interaction between body posture and the order in which the experiment was conducted on perceived duration. This interaction effect indicated that participants tended to under-produce time in the chair in which they started the experiment, regardless the type of chair they were sitting in, and therefore, of arousal. This suggests a learning effect during the course of the experiment: participants became more accurate. It also further supports the finding that arousal did not affect perceived durations.

Finally, we found an effect of pleasure on perceived duration in the relaxing chair. The more pleasant participants felt in the relaxing chair, the shorter the time productions and thus the longer duration estimates. This result is in accordance with the results of Angrilli et al. (1997). They also found overestimation of durations when a low arousal positive picture was shown. Pleasure could not explain the odor effect on perceived duration, therefore, it should be considered to be an additional factor influencing subjective time duration.

The results of this study have generated new insights in external factors that can influence perceived duration in prospective timing. To our knowledge, this is the first study showing that odor can shorten time productions. We also found that this odor effect was not caused by arousal. The findings contribute to the current literature on perceived duration as we showed that for longer durations the arousal mechanism of the internal clock model does not apply. Other mechanisms were proposed but remain to be fully investigated in future research.

## Author contributions

The literature review and experiment were conducted by Eliane Schreuder. Eliane Schreuder wrote the first draft of the article. Marco R. Hoeksma, Monique A. M. Smeets, and Gün R. Semin supported with the experimental design, reviewed and re-edited the final article.

### Conflict of interest statement

The authors declare that the research was conducted in the absence of any commercial or financial relationships that could be construed as a potential conflict of interest.

## Footnotes

^1^We focussed on cognitive models concerning timing of longer time scales such as seconds, minutes and months, as timing of shorter or automatic time scales (tens to hundreds of milliseconds) appears to be underpinned by different neural mechanisms (Buhusi and Meck, 2005; Eagleman and Pariyadath, 2009).
